# *Toxoplasma gondii-*Induced Long-Term Changes in the Upper Intestinal Microflora during the Chronic Stage of Infection

**DOI:** 10.1155/2018/2308619

**Published:** 2018-11-01

**Authors:** Emese Prandovszky, Ye Li, Sarven Sabunciyan, Curtis B. Steinfeldt, Lauro Nathaniel Avalos, Kristin L. Gressitt, James R. White, Emily G. Severance, Mikhail V. Pletnikov, Jianchun Xiao, Robert H. Yolken

**Affiliations:** ^1^Stanley Division of Developmental Neurovirology, Department of Pediatrics, Johns Hopkins School of Medicine, Blalock Bldg. 600 N. Wolfe St., Baltimore, MD 21287, USA; ^2^Resphera Biosciences LLC, 1529 Lancaster Street, Baltimore, MD 21231, USA; ^3^Department of Psychiatry, Johns Hopkins University School of Medicine, Baltimore, MD, USA

## Abstract

*Toxoplasma gondii* is an obligate intracellular parasite with worldwide distribution. Felines are the definitive hosts supporting the complete life cycle of *T. gondii.* However, other warm-blooded animals such as rodents and humans can also be infected. Infection of such secondary hosts results in long-term infection characterized by the presence of tissue cysts in the brain and other organs. While it is known that *T. gondii* infection in rodents is associated with behavioral changes, the mechanisms behind these changes remain unclear. Alterations of the host intestinal microflora are recognized as a prominent role player in shaping host behavior and cognition. It has been shown that acute *T. gondii* infection of mice results in microflora changes as a result of gastrointestinal inflammation in inbred mouse models. The long-term effects of chronic *T. gondii* infection on microbial communities, however, are unknown. In this study, after we verified using our model in terms of measuring microflora changes during an acute episode of toxoplasmosis, we assessed the microbiome changes that occur during a long-term infection; then we further investigated these changes in a follow-up study of chronic infection. These analyses were performed by constructing and sequencing 16S rRNA amplicon DNA libraries from small intestine fecal specimens. We found that acute infection with the GT1 strain of *T. gondii* caused an enrichment of Bacteroidetes compared with controls in CD1 mice. Strikingly, this enrichment upheld throughout long-term chronic infection. The potential biological consequences of this alteration in rodents and humans should be subjected to further exploration.

## 1. Introduction

*Toxoplasma gondii (T. gondii)* is a common food- and water-borne parasite of the phylum Apicomplexa. This organism has been found to infect warm-blooded animals, including humans [1, 2]. Following acute infection, which is often asymptomatic in immune competent hosts, *Toxoplasma* enters a period of long-term infection characterized by altered parasite metabolism and the formation of tissue cysts, largely in the brain and muscles [3]. Chronic infection has been associated with behavioral changes in rodents, with infected rodents showing hyperactivity and decreased predator awareness [4–6]. In humans, *Toxoplasma* seropositivity correlates with behavioral changes [7, 8], prevalence of schizophrenia [9–11], traffic accidents [12, 13], and suicide attempts [10, 14, 15]. Several mechanisms have been proposed for *T. gondii*-mediated behavioral changes, including alterations in neurotransmitter levels [4, 16–18] and immune activation [19]. However, the causal mechanism still needs to be resolved.

Alterations in microflora are increasingly being recognized as a potential mechanism for shaping host behavior and cognition. Recent studies indicate that rodent behavior can be affected by changes in the intestinal microbiome [20–25]. It has been reported that infections with other parasites, such as *Trichuris muris*, can lead to anxiety-like behavior changes, which can be normalized by treatment with a probiotic strain, *Bifidobacterium longum* [26]. These studies suggest that alteration of the intestinal microflora should be considered during *T. gondii* infection. While acute *T. gondii* infection has been used as a tool to induce inflammatory bowel disease in mice with expected microbiome changes [27], the long-term consequences of *T. gondii* infection on the intestinal microflora are unknown. Thus, after use of the adapted mouse model was verified in terms of measuring microflora changes during an acute episode of toxoplasmosis, the microbiome changes that occur during a long-term infection were assessed; then the effect of chronic infection with *T. gondii* on the intestinal microbiome was investigated in a larger independent cohort.

## 2. Method

### 2.1. Mouse Infection

All experimental protocols, including *T. gondii* infection and anesthesia, were approved by the Animal Care and Use Committee at Johns Hopkins University. We tested two mouse cohorts to assess microbiome changes in the upper intestinal tract. For the first cohort, twenty CD1 outbred mice were used to verify using our adapted mouse model in terms of measuring microflora changes during *T. gondii* infection. Out of 10 mice comprising the acute group, 5 mice were infected intraperitoneal (IP) with 500 *T. gondii* tachyzoites, strain GT1, in phosphate buffered saline (PBS), at 6–8 weeks of age after a 3-day acclimation period upon arrival. Five control mice of the same age were inoculated with PBS by the IP route. All 5 infected mice were housed together, while all 5 of the uninfected mice were housed together in a separate cage. The acute group was sacrificed at 5 days postinfection (dpi). Out of the 10 mice comprising the first chronic cohort, 5 were infected IP with 500 GT1 tachyzoites in PBS at 6–8 weeks of age after a 3-day acclimation period. Five control mice of the same age were inoculated with PBS by the IP route. For both the chronic infected and uninfected groups, 3 of the 5 mice from each group were housed individually, while the remaining 2 mice from each group were pair-housed. The chronic group was sacrificed at 5 months postinfection when behavior tests were completed. Mice in the chronic group (both control and infected groups) were treated with sulfadiazine sodium (Sigma-Aldrich, St. Louis, MO) in water from day 5 to day 30 postinfection to control acute infection with the highly virulent GT1 strain and ensure survival. A total of 62 CD1 mice comprised the follow-up second chronic cohort [28], and this main cohort consisted of 17 controls and 45 GT1 infected mice treated as described above for the first chronic cohort.

Mice were anesthetized and humanely sacrificed. Abdominal cavities were dissected, intestines were unfolded, and the distal jejunum and ileum were identified using the ileocecal valve as a landmark. Fecal content (jejunal-ileal fecal content, JIFC) was gently squeezed out from the abovementioned areas, and then the intestine was flushed with PBS. The flow-through containing JIFC was collected into a microcentrifuge tube and kept on ice. Prior to freezing at −80°C, the pellet and supernatant were separated by centrifugation at 10,000 rpm for 5 min.

In addition, blood and spleen from each mouse were obtained to assess the state of the infection in the first cohort. Infection was confirmed in the acute group by the measurement of *T. gondii* 5S rRNA in the spleen by means of a quantitative PCR reaction based on 3 independent assays, using 100 ng/*μ*l spleen DNA from each sample for each reaction (TaqMan® Gene Expression Assays, Life Technologies, Carlsbad, CA). All available spleen tissue was homogenized to avoid sampling error, and the homogenate was processed for DNA isolation using the MoBio Ultraclean Tissue & Cell DNA isolation kit (MoBio, 12334-250, Carlsbad, CA). In the chronic group, infection was documented by the measurement of IgG antibodies to *Toxoplasma* whole cell organism following the manufacture's protocol (Vir-ELISA Anti-Toxo kit, VIRO-Immun Labor-Diagnostika GmbH, Germany, EG 127) (Table S2). In the second chronic cohort, to assess the state of infection *Toxoplasma* whole cell organism, IgG and MAG1 IgG assays were run as described previously [28] (Table S8).

### 2.2. Library Preparation

Genomic DNA was extracted from JIFC with a commercially available DNA isolation kit (QIAmp DNA Stool Mini kit, Cat# 51504, Qiagen, Venlo, Netherlands—acute group; ZR Fecal DNA Mini Prep, Cat # D6010 Zymo, Irvine, CA—chronic group). The quality and the quantity of the nucleic acids were determined by the TapeStation (Agilent Technologies, Santa Clara, CA) bioanalyzer. The entire bacterial 16S rDNA (1.5 Kb) was amplified using 20 ng input DNA in each PCR reaction, utilizing a modified protocol, which was based on previously described methods [29]. The 1.5 kb amplicons were extracted from agarose gel by centrifugation, and the DNA was sheared to 200–300 bp fragments employing a Bioruptor (Covaris, Woburn, MA) by applying a previously determined minimal cycle number (10–12 cycles). Twenty individually indexed libraries were prepared following the manufacturer's instructions (TruSeq ChiP kit, Cat# IP-202-1012, Illumina, San Diego, CA). Each library was quantified using the SYBR green-based KAPA Illumina library quantification kit (KAPA Biosystem, Wilmington, MA), and concentration was normalized to 4 nM.

To assess the bacterial composition of the mice in the second chronic cohort, JIFC was similarly collected from the small intestine for 16S rRNA gene sequencing. To control for environmental contaminants, blank and library preparation control samples were also included in the library preparation and sequencing process. Genomic DNA was isolated from infected and uninfected animals, using Zymo ZR Fecal Mini kit. Individually indexed libraries were prepared targeting the v3-v4 region on the 16S rRNA gene using the standard Illumina 16S metagenomic library preparation guide. Each library concentration was determined using Qubit normalized to 4 nM.

A 5 *μ*l aliquot of each library was pooled and denatured using 0.2 N NaOH and subsequently diluted to 10 pM using HT1 (hybridization buffer) supplied by Illumina (San Diego, CA). Prior to loading, the library was spiked with a 10% 20 pM PhiX control. Sequencing was performed on the MiSeq Illumina sequencing platform using a 2 × 300 PE sequencing reagent kit (Cat # MS-102-3003 Illumina, San Diego, CA).

#### 2.2.1. Sequence Analysis with QIIME 1

Raw paired-end reads were first demultiplexed using 5′ index information, trimmed of adapter sequences, and screened for PhiX174 contaminating sequences. To combine paired-end reads, FlasH command line tool (http://ccb.jhu.edu/software/FLASH/) was utilized [30]. Using the Quantitative Insights Into Microbial Ecology (QIIME) package (v.1.8.0) [31, 32], reads were filtered for quality by trimming sequences located after a five consecutive low-quality base call (Phred score < 20) and by requiring a final trimmed sequence length of at least 150 bp.

To maximize sequencing depth per sample, targeted V3–V5 sequence analysis was performed by first screening for reads that matched the v3 (357F) primer sequence to within one nucleotide mismatch. Reads matching the primer within 60 bp of the 5′ end were trimmed to the end of the primer match. The resulting high-quality read set was then screened for chimeric sequences with UCHIME (*de novo* mode) [33], as well as chloroplast and mitochondria DNA using the Ribosomal Database Project classifier [34]. A final BLASTN search was performed against the Greengenes 16S database (v13_5) to identify contaminants. Sequences without a database match of at least 70% identity along at least 50% of their length were removed from downstream processing.

Remaining sequences were clustered into operational taxonomic units (OTUs) using UCLUST [35] with a 95% identity threshold. Representative sequences of each cluster (reflecting the most abundant sequence) were assigned to a taxonomic lineage by the Ribosomal Database Project classifier [34], trained on a custom version of the comprehensive SILVA 16S database [36] using a minimum confidence threshold of 0.50.

Representative sequences were input into PyNAST [37] to generate a multiple sequence alignment, which was subsequently used to construct a neighbor-joining phylogenetic tree with FastTree [38]. Prior to downstream statistical analysis, samples were rarefied [39] to obtain even coverage. Alpha and beta diversity metrics were computed in QIIME. Additional statistical analyses were performed in *R* (v.3.0.0). Tests of differentially abundant taxa, due to the small sample size, employed the negative binomial test implemented within the DESeq package [40], while differential alpha diversity testing utilized Welch's *t*-test. A value of *p* < 0.05 was considered to indicate significant differences between groups and a value of 0.05 < *p* < 0.1 to indicate a suggestive difference. *Q* values were *p* values, corrected for the performance of multiple comparisons by employing false discovery rate (FDR) adjustment.

#### 2.2.2. Sequence Analysis with QIIME 2

Raw paired-end reads were first demultiplexed using 5′ index information, and adapter sequences were trimmed off prior access. R1 and R2 reads were analyzed separately using the QIIME 2 (v 2017.12) platform [41] to preserve sequence information that would have been discarded due to poor overlap between R1 and R2 reads. In addition to filtering out any remaining PhiX contaminants and chimeric sequences, the DADA-q2 plugin [42] was utilized to remove low-quality reads. In this process, R1 and R2 reads were truncated to 158 bp (QS 28) and 133 bp (QS 32) high-quality read lengths, respectively. Since the quality filtered R1 reads were slightly longer, the R1 analyses were focused upon in this report. All unique sequences were collected into a feature table, which was filtered for any potential contaminants that were amplified in blank DNA extraction samples (*n*=12) and low-abundance features using the feature-table q2 plugin [43]. Next, multiple sequence alignment was performed by employing the MAFFT alignment plugin [44] followed by computing a phylogenetic tree using FastTree program [45]. Phylogenetic diversity analyses utilized this phylogenetic tree in order to calculate several alpha and beta diversity metrics.

To explore the bacterial composition of the samples, taxonomy was assigned to sequences using a pretrained naive Bayes classifier and q2-feature-classifier plugin (https://github.com/qiime2/q2-feature-classifier). This classifier was trained on Greengenes v13_8 99% OTUs, where the sequences have been trimmed to only include v3-v4 regions of the 16S rRNA gene bound by Illumina primer pairs. Differential abundance was assessed by utilizing the ANCOM plugin [46], where W-statistic computes a value for each taxon, which describes the number of taxon that a single taxon is tested to be significantly different against. The result of W-statistic was concluded in a hypothesis test, where the null hypothesis was the lack of significant difference between the compared groups in each individual taxon. For statistical analyses, QIIME 2 built-in packages were used. Visualization was generated using MS-Excel and QIIME 2 View.

## 3. Results

### 3.1. Acute *T. gondii* (GT1 Strain) Infection Alters Intestinal Microbiome in CD1 Mice

To define the diversity and the composition of intestinal microflora in acutely infected mice, 16S rDNA libraries, derived from small intestinal JIFC specimens of 5 infected and 5 uninfected control mice, were sequenced. Sequences were analyzed utilizing the Quantitative Insight Into Microbiological Ecology (QIIME) pipeline focusing on the v3-v4 region. Following quality and content (chimeric sequences, mitochondria, and chloroplast) screening (Table S1), a total of 193,593 reads were obtained from the 10 mice in the acute group, 5 of which were infected with *T. gondii* and 5 of which were controls. Following normalization and rarefaction, each sequence set consisted of an average of 6,116 reads per mouse.

**Figure 1:**
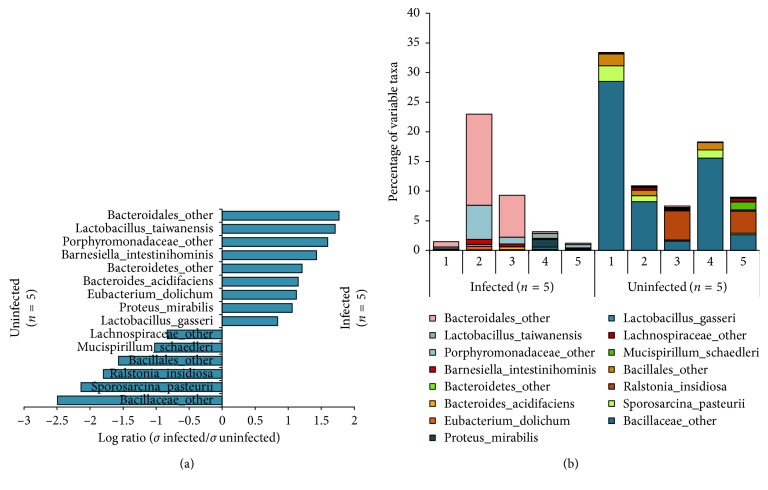
Individual differences at species level in acute *T. gondii*-infected and healthy mice of the first cohort. 5 CD1 mice were infected IP with 500 *T. gondii* tachyzoites (GT1) in PBS, along with 5 control CD1 mice by the IP route with PBS only. The group was sacrificed at 5 dpi. Whole 16S rDNA libraries were sequenced using the MiSeq Illumina sequencing platform to profile small intestinal microbiome. Sequences were analyzed using QIIME pipeline focusing on the v3-v4 region. The graph shows (a) the log-transformed ratios of averaged standard deviation of the relative abundance of highly variable species in infected and control mice and (b) the percentage of highly variable species of the upper intestinal microflora in each mouse. Lactobacillus, Proteus, and Bacteroidetes species are increasingly unstable in the acute infected mice, while Bacillaceae species appear unstable in the uninfected mice.

Taxonomically, the following six bacteria phyla were identified: Firmicutes, Bacteroidetes, Proteobacteria, Verrucomicrobia, Actinobacteria, and Deferribacteres. Firmicutes was the most abundant phylum. Bacteroidetes was the only phylum that showed significant changes related to acute *Toxoplasma* infection, with the prevalence of this phylum significantly increased in infected animals (Table 1, Table S3). At the species level, high individual differences in species richness were found between mice. There was not one single species that consistently enriched in either the control or the infected groups. Rather, each individual mouse had its own erratic species composition. As a result of acute infection, these altered species were well represented in both infected and uninfected groups. In the acute uninfected group, Bacillaceae species appeared especially highly variable, while in the acute infected group, Lactobacillus, Proteus, and an unspecified Bacteroidetes genus appeared to be inconstant (Figure 1, Table S5).

### 3.2. Chronic *T. gondii* Infection Alters the Diversity of the Intestinal Microbiome

#### 3.2.1. First Chronic Cohort

To define the diversity and the composition of intestinal microflora in chronically infected mice, 16S rDNA libraries, derived from small intestinal JIFC specimens of 5 infected and 5 uninfected mice were sequenced. Sequences were analyzed utilizing the QIIME pipeline focusing on the v3-v4 region. Following quality and content screening (Table S1), a total of 345,050 valid reads were obtained from the mice chronically infected with *T. gondii* and their corresponding controls. Following normalization and rarefaction, each sample consisted of an average of 25,887 reads per animal.

**Figure 2:**
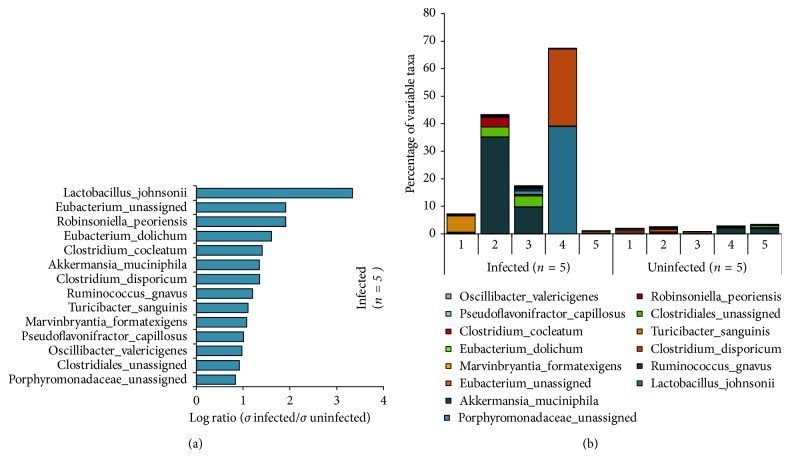
Individual differences at the species level in chronic *T. gondii*-infected and healthy mice of the first cohort. CD1 mice were infected IP with 500 *T. gondii* tachyzoites (GT1) in PBS, along with 5 control CD1 mice by the IP route with PBS only. The group was sacrificed at 5 mpi. Whole 16S rDNA libraries were sequenced using the MiSeq Illumina sequencing platform to profile small intestinal microbiome. Sequences were analyzed using QIIME pipeline focusing on the v3-v4 region. The graph shows (a) the log-transformed average of highly variable species in infected and control groups and (b) the percentage of highly variable species of the upper intestinal microflora in each mouse. Highly variable species only appeared in the infected mice and the vast majority of them belonged to the Firmicutes phyla.

The most abundant phylum was the Firmicutes. Additional 4 phyla were detected including Proteobacteria, Bacteroidetes, Actinobacteria, and Verrucomicrobia. The relative abundance of the Verrucomicrobia phylum was significantly increased (*p* < 0.01, *q*=0.01) in animals with chronic *T. gondii* infection. There was also a trend towards increased levels of Bacteroidetes (*p*=0.08, *q*=0.14). There were no phyla that were significantly decreased in the chronically infected animals as compared with controls (Table 1; Table S4). Interestingly, species level analyses showed random species that only appeared to be part of the microflora of the chronically infected group, whereas the chronic uninfected group was found lacking these random species. The majority of these erratic species in the chronically infected group belonged to the order Clostridiales, while the most variable one, *Lactobacillus jhonsonii*, is a member of the order of Lactobacillales (Figure 2; Table S6).

To estimate species richness within a sample group, alpha diversity was calculated using the Shannon index and the number of operational taxonomy units (OTU). The Shannon index is a commonly used diversity index that takes into account both relative abundance of species and population evenness in the community. OTU is defined as a cluster of reads with 95% similarity, which is used for classifying microbes with the expectation that these OTUs correspond approximately to species.

**Figure 3:**
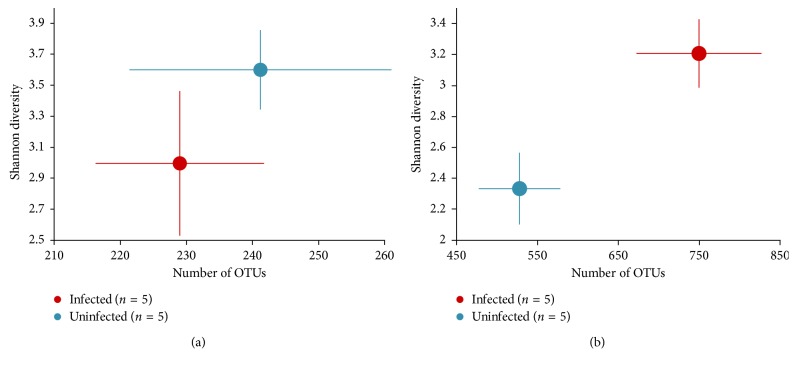
Alpha diversity. Alpha diversity was computed using the Shannon index and average number of OTU. In the first cohort, alpha diversity in CD1 mice infected with *T. gondii* is statistically similar compared with uninfected mice in the acute group while enriched in the chronically infected mice (*p* < 0.05, Welch's *T*-test) compared with corresponding controls. (a) Acute; (b) chronic.

**Figure 4:**
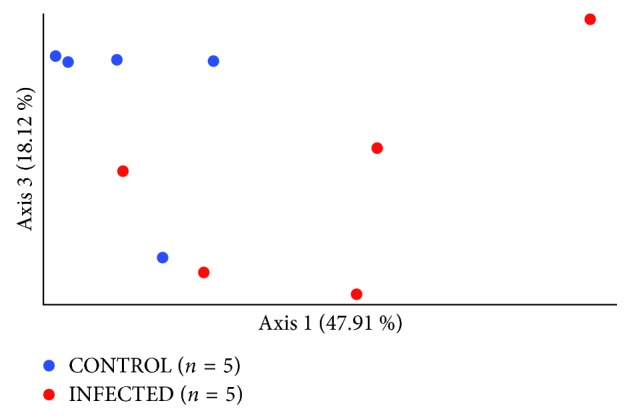
Beta diversity—Weighted UniFrac distance matrix represents dissimilarities between the uninfected control (*n*=5) and chronically infected mice (*n*=5). Pairwise Permanova test was used to calculate the level of significance between the two groups, CONTROL vs. INFECTED (*p*=0.03).

In Figure 3, alpha diversity was calculated by plotting the Shannon index and the average number of OTU, which revealed that the alpha diversity was greater in the chronically infected group (*p* < 0.05, Welch's *T*-test) compared with the corresponding control. However, the alpha diversity in the acutely infected mice did not differ from that of the control. To estimate dissimilarities in the community, Weighted UniFrac distance was computed (Figure 4.). As the principal coordinate plot depicts in Figure 4, controls were clustered close to each other while infected samples were widely spread out. Pairwise Permanova test revealed a significant difference between the control and infected mice in the first chronic cohort (*p*=0.03, *q*=0.03).

#### 3.2.2. Second Chronic Cohort

A second cohort was introduced to confirm the changes seen in the upper intestinal microflora of the smaller cohort of chronically infected outbred mice. Out of 62 CD1 mice, 45 were infected with the *Toxoplasma gondii* GT1 strain, while 17 were inoculated with PBS by the IP route. Sequences were processed using the upgraded QIIME 2 pipeline. After denoising and content filtering, a total of 458,793 valid reads were obtained from the mice chronically infected with *T. gondii* and the control mice, where the average frequency was 7,399 reads per mouse. The subsequent analyses were performed on the rarefied sample set where each sample contained 2,016 reads (Table S7).

**Figure 5:**
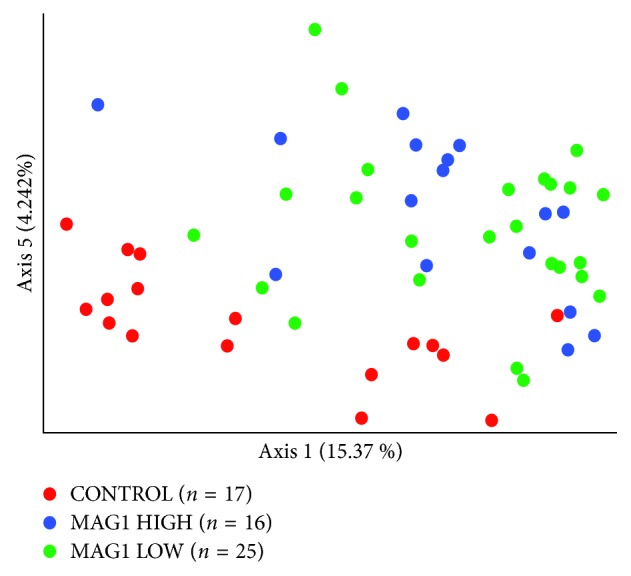
Beta diversity—Jaccard distance. Holding the lowest *p* values, Jaccard distance matrix was selected to represent dissimilarities between uninfected control (*n*=17), MAG1-HIGH chronically infected mice (*n*=16), and MAG1-LOW chronically infected mice (*n*=25) subgroups. Pairwise Permanova test was used to calculate the level of significance between subgroups, MAG1-HIGH vs. MAG1-LOW (*p*=0.04), CON vs. MAG1-HIGH (*p*=0.003), and CON vs. MAG1-LOW (*p*=0.001).

Community richness, measured by four different alpha diversity metrics (observed species, Shannon's, Faith's phylogenetic diversity, and Pileou's Evenness indexes), was comparable between the control and infected mice (*p*=0.25, *p*=0.7, *p*=0.69, and *p*=0.91, respectively; Kruskal–Wallis pairwise test). However, the community composition was significantly different between the control and infected mice (*p*=0.001, *q*=0.001; *p*=0.003, *q*=0.003; *p*=0.009, *q*=0.009; *p*=0.018, and *p*=0.018; pairwise Permanova test) when measured by all of the four beta diversity metrics (Jaccard distance, Bray–Curtis distance, Unweighted UniFrac distance, and Weighted UniFrac distance, respectively) (Figure 5). The beta diversity results were verified by ANCOM analyses that indicated the two taxa that were significantly different between the controls and infected mice (W-test, rejected null hypotheses) were Lactobacillales and the Bacteroidales.

**Figure 6:**
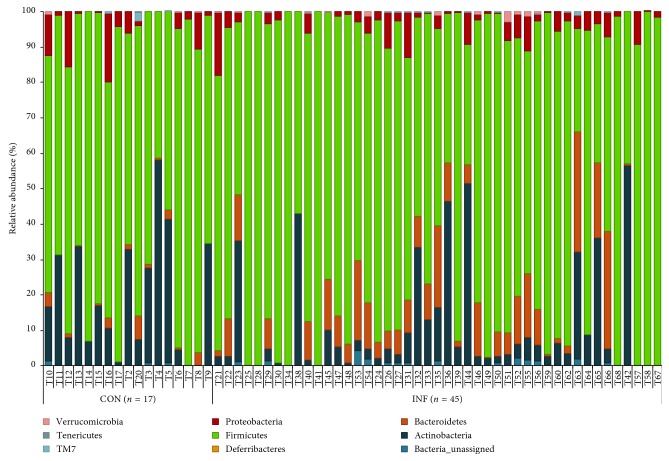
Individual differences at phylum level in chronically *T. gondii* infected and healthy mice of the second cohort. 45 CD1 mice were infected IP with 500 *T. gondii* tachyzoites (GT1) in PBS, along with 17 control CD1 mice by the IP route with PBS only. The group was sacrificed at 5 mpi. Whole 16S rDNA libraries were sequenced using the MiSeq Illumina sequencing platform to profile the small intestinal microbiome. Sequences were analyzed using QIIME 2 pipeline focusing on the v3-v4 region. The graph shows enrichment of Bacteroidetes phylum in chronically infected mice.

Parasite cyst abundance in the brain is reflected by level of IgG directed against the *T. gondii* surface antigen, MAG1 [28, 47]. After breaking down the infected group to MAG1-HIGH (*n*=16) and MAG1-LOW (*n*=25) subgroups (Table S8), beta diversity analyses revealed discrete clustering between the two subgroups in two qualitative community dissimilarity metrics, Jaccard distance, and Unweighted UniFrac distance (Figure 5, Table 2). However, ANCOM analyses did not highlight any particular taxa that would be responsible for this trend. Individual variation at the phylum level is depicted in Figure 6 (Table S9).

## 4. Discussion

In this study, we examined if there are differences in the bacterial communities of the host small intestine in our adopted mouse model following acute and chronic *T. gondii* infection and further examined changes in the gut microbiome in a larger study of mice with long-term infection. The findings from the acute cohort replicated those of others in terms of increased abundances of the Bacteriodetes at the expense of decreased abundance of the Firmicutes. In the chronic infection model, microbial patterns were found that were both distinct and similar to those of acute infection. Most notably, chronic infection was also strongly characterized by an enrichment of the Bacteroidetes phylum at the expense of decreased abundance of the Firmicutes (Lactobacilli). Furthermore, community dissimilarity assessment revealed that upper intestinal bacterial community clusters were associated with parasite cyst abundances. Overall, our investigation represents an initial inquiry into the complexities of the gut microbiome and introduces a number of novel tools to further examine how *T. gondii* infection affects gut microbial dynamics.

While acute *Toxoplasma* infection-induced microbiome changes are well characterized in inbred mice, to date, this is the first study to examine alterations to the gut microflora of outbred mice following long-term *T. gondii* infection. Here, we established a model that could serve as a tool to shed light on potential underlying mechanisms of ocular toxoplasmosis and encephalitis caused by a type-1 toxoplasma infection common in immunocompromised patients in the United States and Brazil. In contrast to many other mouse studies, a less artificial model was introduced, where a genetically more diverse outbred mouse strain (CD1) was infected with a more virulent type-1 strain of *T. gondii* (GT1), which has not been adapted to the laboratory environment as long, to explore the potential changes in the microflora and behavior [28]. Furthermore, in our experimental design, the IP route was used for infection in order to avoid the detrimental effect of orally administered parasites on the intestinal epithelium and to utilize the same route of delivery most commonly used for behavior experiments. The small intestine was selected for analyses because the small intestine is where the majority of host-microbiome interactions take place [48]. The v3-v5 region of bacterial 16S rDNA was also focused upon because this portion provides the most accurate characterization of a wide range of host bacteria [49]. This adapted model was then verified by studying the effect of acute *T. gondii* infection on intestinal microflora found previously.

Our findings for this model are consistent with previously published results of acute infection with *T. gondii*. Several studies have examined the effect of oral infection (gavage) with *T. gondii* on the intestinal microbiome of inbred mice. All of these studies employed oral administration of the Me49 strain to C57BL/6 mice and characterized the microbiome during acute infection [50–54]. Previous studies have shown that during acute *Toxoplasma* infection, three major microbial phyla are affected: Firmicutes, Proteobacteria, and Bacteroidetes. The effects of acute *Toxoplasma* infection on the microbiome observed in this study using outbred mice were similar to the effects reported with other models in which increased numbers of Bacteroidetes [50–54] and decreased levels of Firmicutes [51, 52, 54] were identified. These similarities indicate that changes in these phyla following acute infection may occur despite differences in the infecting *Toxoplasma* strain, the route of infection, or the host mouse strain. However, increases in Enterobacteriaceae (Proteobacteria) were not identified in this study, but have been reported in other models of acute *Toxoplasma* infection [51–54]. These differences could be due to the different routes of infection, the different mouse strains [55], or the different types of *T. gondii* strains employed in the distinct experimental models.

Following the resolution of acute infection, *Toxoplasma* can establish a chronic infection characterized by tissue cysts in the brain and other organs, as well as altered behavior and decreased levels of cognitive functioning [28]. Previously published studies, to the best of our knowledge, have not examined the effect of chronic *Toxoplasma* infection on the microbiome. Thus, the small intestinal microflora of chronically infected mice was assessed along with age-matched controls in two independent experiments. Strikingly, significant differences were found in the microflora of chronically infected mice, even after 5 months postinfection, compared with the control mice. Similar to the acutely infected group, the most prominent differences were found in the Bacteroidetes phylum, which was significantly enriched in the chronically infected animals in the second cohort while showing trend of increase in the first cohort.

Significantly increased species diversity in the chronically infected group was observed compared with the controls in the first cohort; however, such differences were not detected in the second cohort. This may have been the result of shorter read lengths, since only reads from one end were taken into account, which can influence the accuracy of species level identification [34, 56]. On the contrary, in the acute infection model, in contrast to previous findings [50, 51], such differences were not detected in species richness. This could, again, be due to differences in the route of infection, mouse strains [55], or types of *T. gondii* strains employed in the different experimental models.

Beta diversity analyses revealed significant differences in community composition between the chronically infected and control mice in both cohorts. Moreover, ANCOM analyses indicated that the two taxa that were significantly different between the control and infected mice were Lactobacillales and the Bacteroidales in the second cohort. Concentration of MAG1 in the infected animals blood is correlated with cyst abundance [28]. To observe whether cyst abundance shows any correlation with certain bacterial taxa, the infected group was broken down to MAG1-HIGH and MAG1-LOW subgroups and beta diversity analyses were performed in the same chronically infected cohort. Two qualitative community dissimilarity metrics, where abundance is not taken into account, revealed significantly distinct clustering between the two subgroups. However, these distinct clusters were not associated with any particular taxa based on the ANCOM analyses, which can be the caused by the loss of statistical power due to breaking down the infected group into subgroups.

Behavior and cognitive functioning was directly measured in the mice that were analyzed for microbiome composition in this study [28]. Previous studies in mouse models have shown that alterations in the intestinal microbiome are associated with changes in behavior and cognitive functioning. Many of these studies have focused on Lactobacilli and other Firmicutes [22, 23, 25]. Some of the same taxa were affected by *T. gondii* infection in the upper gastrointestinal tract of chronically infected mice in this study. This finding suggests that during the chronic stage of *T. gondii* infection, changes in the relative abundance of Firmicutes and Bacteroidetes may play an important role in shaping the host behavior long after the initial acute infection had been resolved. *Toxoplasma gondii* infection in humans has been associated with a number of neurological disorders and cognitive changes, including schizophrenia [57, 58]. The exact mechanisms underlying these behavior changes still remain unclear, yet it is noteworthy that alteration of the intestinal microflora has been reported in recent onset psychosis in humans [59]. The potential biological consequences of the upper microflora alteration in humans during chronic *T. gondii* infection should be subject to further exploration.

This study had some limitations. No attempts were made to further manipulate the microbiome in the *Toxoplasma*-infected mice. Follow-up studies on microbiome changes, including additional behavior and manipulation experiments related to chronic infection, with different *Toxoplasma* and mice strains, will be pursued. Furthermore, the intestinal microbiome was measured at only two time points. Since many behavioral abnormalities have neurodevelopmental features with a critical window, it will be important to assess microbiome changes at additional time points throughout development.

Despite these limitations, this is the first study to document that *Toxoplasma* infection leads to long-term changes in the intestinal microbiome. In particular, these changes in the intestinal microbiome involve the emergence of an increased number of Bacteroidetes in infected animals. In addition, the types of bacteria showing significantly altered levels are similar to those associated with changes in behavior and cognition. Further studies are necessary to conduct additional analyses of the interaction between *Toxoplasma* infection, the intestinal microbiome, and behavior in mice and other species, including humans. These studies may lead to an increased understanding of the mechanisms by which *Toxoplasma* infection alters brain functioning, and subsequently, improved methods for the amelioration of these effects in affected individuals.

## Figures and Tables

**Table 1:** Relative abundance of each phyla in acute group and the first chronic cohort.

Phylum	Acute				Chronic			
	Uninfected^a^	Infected^a^	*p* value^b^	*q* value^c^	Uninfected^a^	Infected^a^	*p* value^b^	*q* value^c^
Bacteroidetes	0.19	6.81	<0.01	<0.01	0.24	0.74	0.08	0.14
Firmicutes	86.04	64.57	0.15	0.25	99.04	98.06	0.06	0.14
Proteobacteria	8.81	20.02	0.32	0.37	0.36	0.20	0.28	0.33
Verrucomicrobia	3.86	6.44	0.70	0.70	0.01	0.15	<0.01	0.01
Deferribacteres	0.27	0.03	0.11	0.25	ND	ND	ND	ND
Actinobacteria	0.21	0.08	0.18	0.25	0.06	0.09	0.46	0.46
Others	0.63	2.05	0.01	0.03	0.28	0.76	<0.01	0.01

^a^Units displayed as percentage for relative abundance. ^b^Negative binomial test was used to calculate *p* values. ^c^ *q* values were FDR-corrected *p* values.

**Table 2:** Beta diversity metrics in the second cohort (pairwise Permanova).

Metrics type	Group 1	Group 2	Sample size	Permutation	Pseudo-F	*p* value	*q* value
*Bray–Curtis*	MAG1-HIGH	MAG1-LOW	41	999	0.694	0.753	0.753
	MAG1-HIGH	CONTROL	33	999	2.732	0.028	0.042
	MAG1-LOW	CONTROL	42	999	3.047	0.012	0.036

*Jaccard*	MAG1-HIGH	MAG1-LOW	41	999	1.571	0.036	0.036
	MAG1-HIGH	CONTROL	33	999	3.170	0.003	0.005
	MAG1-LOW	CONTROL	42	999	3.804	0.001	0.003

*Unweighted UniFrac*	MAG1-HIGH	MAG1-LOW	41	999	2.871	0.014	0.014
	MAG1-HIGH	CONTROL	33	999	3.374	0.005	0.011
	MAG1-LOW	CONTROL	42	999	3.227	0.007	0.011

*Weighted UniFrac*	MAG1-HIGH	MAG1-LOW	41	999	1.216	0.288	0.288
	MAG1-HIGH	CONTROL	33	999	2.812	0.031	0.047
	MAG1-LOW	CONTROL	42	999	3.116	0.019	0.047

## Data Availability

The sequencing analyses data used to support the findings of this study are included within the supplementary information file. The unprocessed sequencing files (fastq files) used to support the findings of this study are available from the corresponding author upon request.

## Supplementary Materials

Supplementary Materials MS-Excel file (Tables S1–S9) containing metadata information regarding the two animal cohorts along with details on the analyses of the two sequenced 16S rRNA sets is provided.
